# The impact of protective face masks and coverings on patient-health provider communication

**DOI:** 10.1192/j.eurpsy.2021.840

**Published:** 2021-08-13

**Authors:** I. Ganhao, M. Trigo, A. Paixao

**Affiliations:** 1 Clinic 6, Centro Hospitalar Psiquiatrico de Lisboa, Quinta do Anjo, Portugal; 2 Psychology Unit, Centro Hospitalar Psiquiatrico de Lisboa, Lisbon, Portugal; 3 Clinic 4, Centro Hospitalar Psiquiatrico de Lisboa, Lisbon, Portugal

**Keywords:** face masks, face coverings, communication, emotion

## Abstract

**Introduction:**

Amid the COVID-19 pandemic the trend points to universal use of protective face masks. The impact posed on verbal and non-verbal communication by masks is yet another challenge to be addressed in clinical care.

**Objectives:**

To reflect on the consequences of face mask and covering use on communication in the clinical setting, including mental health settings.

**Methods:**

Pubmed and Google Scholar literature search using terms face mask / face covering and communication / emotion.

**Results:**

There is a lack of literature on the impact of protective face masks and coverings on communication in clinical settings. Face masks and coverings may have a significant impact on patient-healthcare professional relationship due to disruption of verbal (poorer quality of speech transmission) and non-verbal communication (emotional expression and recognition) with consequences on: 1) clarity of communication with potential for misunderstanding clinical information, advice and prescriptions posing safety issues, 2) emotion perception, expression and reciprocity, 4) perception of healthcare professionals’ empathy and therefore, 3) patient satisfaction, 4) quality of care, and 5) clinical outcomes. Difficulties in communication between the patients´family or other carers and healthcare providers and between healthcare professionals are likewise challenged. People with hearing impairment, children and people with mental illness may be especially vulnerable to these difficulties in communication.

**Conclusions:**

Protective face masks and coverings are undoubtedly important in preventing spread of COVID-19, nonetheless mental healthcare professionals should take into account their significant impact on verbal and non-verbal communication in clinical care. Alternative strategies to enhance communication and rapport may be warranted.

